# Assessment of Psychological Dimensions in Telemedicine Care for Gestational Diabetes Mellitus: A Systematic Review of Qualitative and Quantitative Studies

**DOI:** 10.3389/fpsyg.2019.00153

**Published:** 2019-02-05

**Authors:** Stefania Fantinelli, Daniela Marchetti, Maria Cristina Verrocchio, Marica Franzago, Mario Fulcheri, Ester Vitacolonna

**Affiliations:** ^1^Department of Psychological, Health, and Territorial Sciences, School of Medicine and Health Sciences, “G. d'Annunzio” University, Chieti, Italy; ^2^Department of Medicine and Aging, “G. d'Annunzio” University, Chieti, Italy

**Keywords:** gestational diabetes mellitus, telemedicine, empowerment, engagement, satisfaction

## Abstract

**Background and Objective:** Gestational Diabetes Mellitus (GDM) is a complex and wide spread problem and is considered one of the most frequent chronic metabolic conditions during pregnancy. According to a recent consensus conference held in Italy, new technologies can play a role in the so-called process of fertilization of the individual's ecosystem engagement, representing support for systemic collaboration among the main actors. The current systematic review aimed at providing an update of the literature about telemedicine for GDM, considering the role of psychological dimensions such as empowerment/self-efficacy, engagement and satisfaction.

**Methods:** The review was performed following the Preferred Reporting Items for Systematic Reviews and Meta-Analyses (PRISMA) framework. The data sources were PubMed, ScienceDirect, Cochrane, and Scopus databases.

**Results:** Thirteen articles were identified as eligible and relevant for the final qualitative synthesis, but none was specific for the topic of engagement. The quality or research bias of the studies presents methodological limits. Most studies had clinical outcomes as a primary object. Concerning empowerment and self-efficacy, there were only preliminary findings reporting any improvements derived from using telemedicine approaches. Conversely, there were more consistent and positive results concerning the satisfaction of patients and clinicians.

**Conclusions:** These results are not sufficient to state a conclusive evaluation of positive effects of telemedicine use for GDM care. A more in-depth investigation of engagement and empowerment dimensions is necessary, as some benefits for the management of chronic conditions were already detected. Further investigations will also be necessary concerning the acceptability and feasibility of telemedicine systems by clinicians.

## Introduction

Gestational Diabetes Mellitus (GDM) is defined as “diabetes diagnosed in the second or third trimester of pregnancy that was not clearly overt diabetes prior to gestation” (American Diabetes Association, 2018). GDM is a particular condition since it is limited in time: indeed, in most cases the pathology regresses after delivery, so that women have a short time available to know and accept their condition. In GDM the mainstay of treatment is medical nutrition therapy with daily self-monitoring of blood glucose. When the blood glucose levels are above those recommended, insulin therapy must be used to minimize the risk to maternal and fetal health (American Diabetes Association, 2018). Furthermore, metabolism changes frequently, so there may be a need for frequent medical visits and therapy modifications, which could cause stress and confusion in an already delicate situation such as pregnancy.

Technological advancements can offer powerful and user-friendly solutions to better cope with chronic conditions. Indeed, many scholars have affirmed that information and communication technologies can enhance a person's condition self-management (Tani, 2010; King, 2012; Guo et al., 2015; Marchetti et al., 2015; Graffigna et al., 2017). Telemedicine can be defined as a subset of telehealth that “uses communications networks for delivery of healthcare services and medical education from one geographical location to another, primarily to address challenges like uneven distribution and shortage of infrastructural and human resources” (Sood et al., 2007, p. 576). “The delivery of health care services, where distance is a critical factor, by all health care professionals using information and communication technologies for the exchange of valid information for diagnosis, treatment and prevention of chronic conditions and injuries, research and evaluation, and for the continuing education of health care providers, all in the interests of advancing the health of individuals and their communities” (WHO Group Consultation on Health Telematics, 1998).

Telemedicine assistance can be useful in GDM for different reasons: (1) to improve the cooperation between women and the care team; (2) because it could provide individuals with worthwhile information about their condition; (3) to improve training to persons and to collect information on their condition; this feature can be a kind of motivational boost and incentive to persons' engagement and acceptance of the chronic condition; (4) to optimize the condition's management. As some authors already depicted (Lee et al., 2015; Haluza et al., 2017), online health information-seeking behavior is a common habit and individuals refer to the Internet as the first and sometimes the only source of information, without a specific reliable reference.

Relevant available information about GDM is important in order to provide knowledge, in particular scientifically validated knowledge and skills that can in turn foster the women's engagement (Graffigna et al., 2017). Individual's engagement in health contexts can have a threefold definition: “understanding the importance of taking an active role in one's health and health care; having the knowledge, skills and confidence to manage health; using knowledge, skills, and confidence to engage in health-promoting behaviors to obtain the greatest benefit” (Simmons et al., 2014, p. 2). Therefore, the knowledge and the informative level can be interpreted as preliminary steps, which lead to engagement.

Telemedicine allows us to overcome the need for physical co-presence. However, as known, it is very difficult to obtain changes in behavior and telemedicine adds a further critical element. Furthermore, self-care and compliance with therapy can be improved by the person's perception of having an always willing clinician even through the telemedicine system. Another dimension often evaluated in relation to telemedicine use is compliance; it has to be said that there are several words to define a similar concept, for example adherence, concordance, or persistence (Vrijens et al., 2012). The main difference is related to the implicit meaning defining the relation between the patient and the physician: compliance implies a passive view of the patient, who should passively accept the physician's prescriptions. Adherence, for example, implicitly describes a more active patient, who collaborates with clinicians (Balkrishnan, 2005). Both terms, compliance and adherence, share the quantifiable dimension related to drug doses; but there is no uniformity in defining and describing adherence and compliance as distinct terms, as highlighted in a review (Vrijens et al., 2012) concerning the taxonomy of adherence to medications. According to the considered papers, compliance in GDM is meant as the women's willingness to follow physicians' prescriptions and suggestions in terms of drug doses or number of insulin measurements.

Another relevant dimension to take into account when assessing the effect of telemedicine care is the satisfaction with care and service; this concept is often described as being strictly related to the met expectations (Batbaatar et al., 2015). Different points of view have defined satisfaction as a multidimensional element (Marcinowicz et al., 2010) including both emotional and cognitive evaluations. These two dimensions are also involved in satisfaction for GDM patients, for example Sayakhot and Carolan-Olah (2016) found that the quality of patient–clinician communication is a key factor affecting the satisfaction with care and service. There are also several studies aimed at evaluating satisfaction with telemedicine systems for GDM, concerning usefulness and perceived ease of use (Jo and Park, 2016; Miremberg et al., 2018).

Thus, the usefulness of a telemedicine system can also reassure the pregnant woman of the possibility of managing GDM rapidly and without wasting money or time. In most cases, telemedicine systems allow persons to record at least their blood glucose data and other parameters and make them available for the health care provider. Several authors have stated that personal health records made the individual focus on positive behavior change (Tang and Lansky, 2005) and in turn motivation and empowerment were improved (Hess et al., 2007). Many authors define empowerment both as an outcome and as a process; patients can be empowered by health education and a patient centered approach or they can empower themselves by seeking information online or having group experiences with other patients (Holmström and Röing, 2009). When talking about empowered women with GDM it means that “the patient should have a clear understanding about the disease, its pathogenesis and both short and long–term consequences for mother and baby” (Ashraf and Hasan, 2018).

There is no doubt concerning the positive role of telemedicine in reducing cost and time for traditional clinical visits and this can, in turn, promote clinicians' commitment. A recent conference consensus on person engagement in the health context (Graffigna et al., 2017) makes a clear reference to the importance of clinicians' commitment, stating that it is determinant to better fostering individual engagement. For this reason, an assessment of clinicians' acceptability of the technology is worth doing in studies aimed at testing the feasibility of telecare systems.

The current systematic review is aimed at identifying research focused on the potential of telemedicine to provide effective diabetes care for women with GDM taking into account the role of psychological dimensions such as empowerment/self-efficacy, engagement and satisfaction. Since clinicians' acceptability of the technology may affect the success of the intervention, a synthesis of this point was accounted for where provided.

Past systematic reviews on the same topic were mostly performed including all diabetes types in pregnancy (Mastrogiannis et al., 2013; Bashshur et al., 2015; Ming et al., 2016), so the present review provides a more in-depth analysis specific for GDM meant as first diagnosis during pregnancy. Other recent systematic reviews analyzed only randomized clinical trials (Bashshur et al., 2015; Rasekaba et al., 2015) focusing just on clinical outcomes and on care costs (Rasekaba et al., 2015; Ming et al., 2016). To the best of our knowledge, there are no review studies to date aimed at evaluating psychological variables in telemedicine for GDM women.

The main research question that motivated our work is concerned with the psychological usefulness of telemedicine for GDM women; can technological help support in improving women's self-efficacy, engagement, empowerment, and satisfaction with care, during GDM?

## Methods

### Information Sources and Searches

A systematic literature review was conducted adopting the Preferred Reporting Items for Systematic Reviews and Meta-Analyses (PRISMA) guidelines (Moher et al., 2009). A comprehensive electronic search strategy was used to identify peer-reviewed articles evaluating the role of psychological dimensions in the implementation of telemedicine systems for GDM care up to June 2018. The search was performed using the following keywords: “gestational diabetes OR gestational hyperglycaemia OR hyperglycaemic pregnancy AND telemedicine OR telecare OR teleassistance OR e-health OR digital healthcare OR m-health AND engagement OR self-efficacy OR empowerment OR satisfaction”.

The databases screened for the literature review included PubMed, ScienceDirect, the Cochrane Library and Scopus.

### Eligibility Criteria

Papers were eligible if they were original research in English or Italian, concerning the effect of telemedicine for GDM, paying attention to an assessment of psychological dimensions such as engagement, empowerment, self-efficacy and satisfaction. Studies evaluating telemedicine only for persons with diabetes (type 1 and type 2) were rejected since our aim was specific for pregnant samples with onset or first recognition of hyperglycaemia during pregnancy. We also excluded papers focused on a description of the technology without any kind of evaluation of its efficacy and acceptability in the target group. We excluded letters to editors, books, books chapters, meta-analyses, reviews, and conference papers. No limit was fixed concerning study design (qualitative or quantitative) or publication date.

### Analysis and Data Synthesis

The selected studies reported different results and they were also varied in terms of sample, design and measures; so, it was not feasible to conduct a proper meta-analysis. We performed a qualitative analysis choosing a narrative approach in order to compare different information from each study. Both qualitative and quantitative studies were included in the analysis to make the evaluation as comprehensive as possible. A qualitative approach can provide more profound knowledge of the topic. Furthermore, when there are mixed methodologies applied, it can be a sort of added value. It is possible to better understand a research object in light of the chosen method, as postulated by the methodological appropriateness paradigm (Patton, 1990).

### Risk of Bias of Studies

Research bias or quality of study from the Cochrane Collaboration Risk of Bias Tool and the Effective Public Health Practice Project Quality Assessment Tool (Armijo-Olivo et al., 2012) were applied to all studies. The risk of bias/quality of research characteristics of each study included whether the method of randomization was described; randomization was concealed at baseline; the trial protocol was preregistered; prior power calculations were performed; assessors were blinded; completeness of follow up reported; selection bias reported; validity or reliability of the measures described; and analysis of possible confounding variables.

## Results

### Study Selection

**Figure 1:**
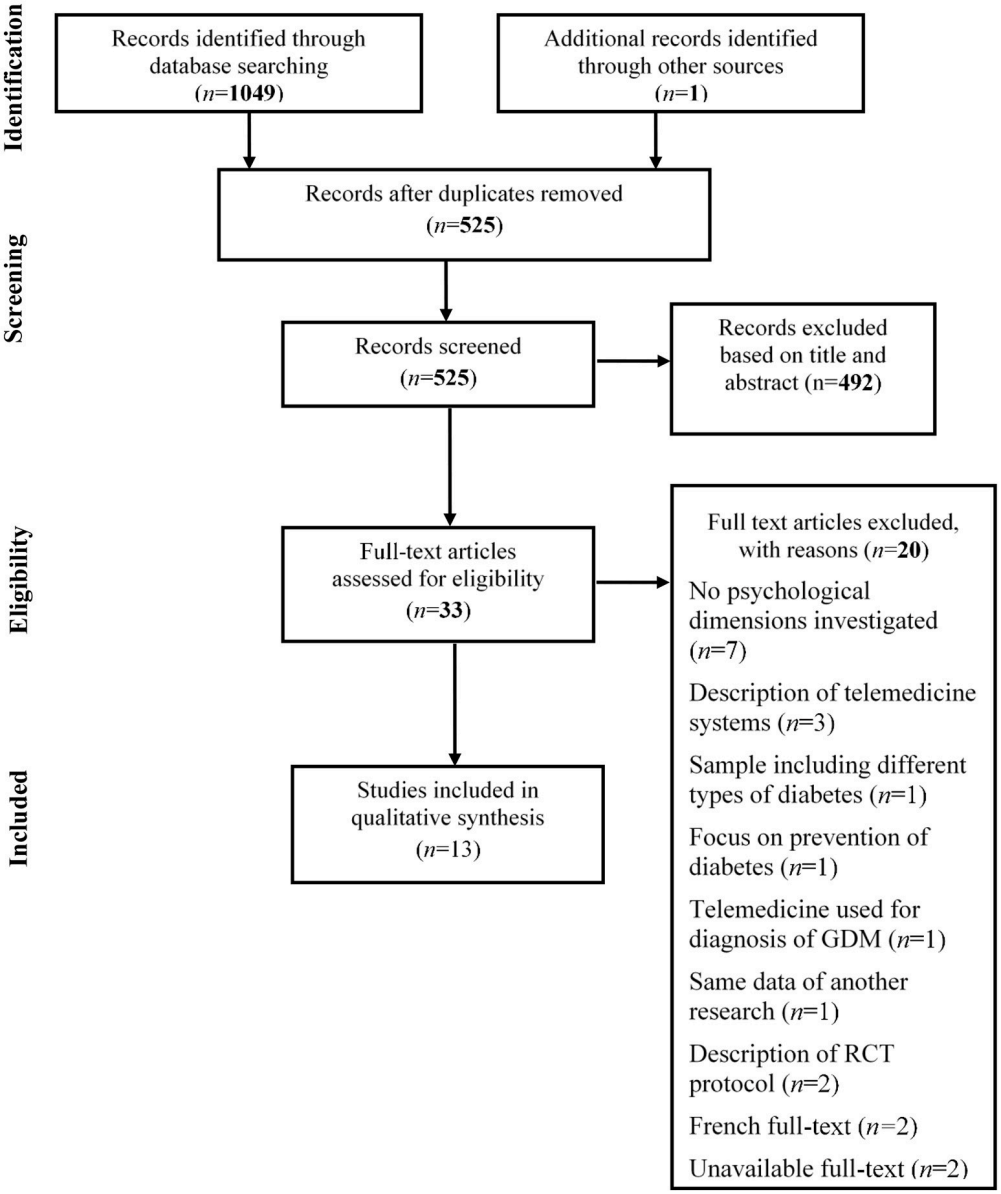
Flowchart of the systematic search.

The search of PubMed, ScienceDirect, Cochrane, and Scopus databases initially produced at first 1050 articles, 33 of which were selected for a full-text screening and 20 were excluded for several reasons (as shown in the PRISMA flow diagram, Figure 1).Thirteen articles were identified as eligible and relevant for the final qualitative synthesis, but none was specific for the engagement topic.

### Quality of Research Within Studies

The quality or research bias of the studies was weak and is displayed in Table 1. Five of the 13 articles (39%) were randomized clinical trials (RCTs). Five (39%) were pure quantitative studies, two (15%) were qualitative studies and one used a mixed-method design with both qualitative and quantitative aspects. The method of randomization was reported by only three (23%) studies; concealment of randomization at baseline was specified by five (39%) studies. Trial preregistration and blinding of assessors were not reported by any of the studies. Only one trial reported statistical power calculations before the conduct of the trial. Completeness of follow up was reported in most of these trials (85%). Selection bias, validity and/or reliability of the measures, and accounting for possible confounders of outcomes were reported by three studies (23%).

**Table 1:** Quality assessment of the selected studies.

**References**	**Method of randomizing reported**	**Randomization at baseline**	**Preregistered trial**	**Prior power calculations**	**Blinding of assessors**	**Completeness of follow-up**	**Selection bias reported**	**Validity/reliability of measure reported**	**Possible confounders reported**
Bartholomew et al., 2015	Y	Y	N	Y	N	Y	Y	N	N
Bromuri et al., 2016	N	N	N	N	N	Y	N	N	Y
Caballero-Ruiz et al., 2017	N	Y	N	N	N	Y	N	N	N
Carolan-Olah et al., 2015	NA	NA	NA	N	N	Y	N	N	N
Dalfrà et al., 2009	NA	NA	NA	N	N	Y	Y	N	N
Given et al., 2015	Y	Y	N	N	N	Y	Y	N	N
Harrison et al., 2017	NA	NA	NA	N	N	Y	N	N	NA
Hirst et al., 2015	NA	NA	NA	N	N	Y	N	Y	N
Homko et al., 2007	N	Y	N	N	N	Y	N	Y	Y
Jo and Park, 2016	NA	NA	NA	N	N	Y	N	Y	N
Kruger et al., 2003	Y	Y	N	N	N	N	N	N	N
Rigla et al., 2018	NA	NA	NA	N	N	Y	N	N	Y
Wickramasinghe and Gururajan, 2016	NA	NA	NA	N	N	N	N	N	N

*N, no, did not meet that item; NA, item not applicable; Y, yes, met that item*.

### Results of Studies

All but three studies (Hirst et al., 2015; Jo and Park, 2016; Harrison et al., 2017) had the evaluation of clinical outcomes related to blood glucose levels and pregnancy outcomes such as delivery, macrosomia, low-birthweight infant, and compliance with care as primary outcomes (see Table 2).

**Table 2:** Features of included studies assessing telemedicine care among GDM women.

**Author (Year) Setting (Country)**	**Study type**	**Aim**	**Number of participants**	**Intervention**	**Control**	**Clinical outcomes**	**Psychological dimensions**	**Measure of psychological dimensions**	**Clinicians' acceptability of the technology**	**Measure of clinicians' acceptability**
Bartholomew et al. (2015) Mathan Kapi'olani Medical Center for Women and Children (Hawaii).	Quantitative study; RCT.	To compare a voicemail system with a cell phone Internet technology (CIT) system for management of hyperglycaemia during pregnancy.	T = 50 Mathan C = 50	Cell phone Internet technology system for recording blood glucose measurement. A confirmation message is provided for data receipt. The physician reviews data on a web-site and provides recommendations by phone. Women contact the physician with any concerns.	Voicemail system for reporting blood glucose measurements.	Compliance with self-monitoring of blood glucose (four times a day).	Women's satisfaction using the system.	Adapted survey of a previous pilot study of the CIT system.	NA	NA
Bromuri et al. (2016) Mathan University Hospital of Lausanne (Switzerland).	Quantitative study; RCT.	To study the feasibility of a Personal Health System to monitor GDM, assessing women's and clinicians' acceptability.	T = 12 Mathan C = 12	Smartphone application to enter and monitor blood sugar measurement, medicine taken by the women and any symptoms related to GDM. The application provides hypoglycaemia alerts both to women and medical staff.	Standard care.	Checking blood glucose with self-monitoring (four times a day).Mathan Number of hypoglycaemic episodes.	Perception of the technology from the perspective of women, caregivers and medical staff.	Qualitative questionnaire.	Doctor perception of benefits for their workload. Opinions, beliefs and attitudes of the healthcare providers.	Qualitative questionnaire.Mathan Focus group.
Caballero-Ruiz et al. (2017) Mathan Parc Tauli University Hospital (Spain).	Quantitative study; RCT.	To describe ‘Sinedie', a telecare and educational health platform for GDM management enhanced by decision support capabilities; to evaluate the system regarding its safety and effectiveness.	T = 60 Mathan C = 30	Web-based system that provides remote persons with GDM monitoring (glycaemia, ketonuria values and diet), automatic data analysis (glycaemic and ketonuria status) and advice on therapy planning. The platform provides notification of the metabolic condition both to women and physicians. A decision support tool for therapy planning is generated combining the person's metabolic condition, previous GDM management and recommendations.	Standard care.	Women's behavior in blood glucose self-monitoring (fasting and postprandial after breakfast, lunch and dinner).Mathan Physician–women interactions (number and duration of face-to-face visits; number and duration of telephone calls).Mathan Clinician workload (time per person).Mathan Decision support effectiveness (number of automatic diet prescriptions; proposals for insulin therapy; number and reason for correction made by physicians to the automatic diet prescription and insulin therapy needs).	Women's satisfaction using the system.	*Ad hoc* questionnaire.	NA	NA
Carolan-Olah et al. (2015) Mathan Sunshine Hospital (Australia).	Quantitative study.	To design and test a website intervention to provide education for women with GDM.	T = 21	Web-based intervention to provide information about GDM definition and consequences for the baby, practical advice and instruction for healthy eating, explanation of the amount and type of physical activity.	NA	Knowledge of GDM, food values and GDM self-management principles.	Person satisfaction with the information on the website.	Knowledge of GDM questionnaire.	NA	NA
Dalfrà et al. (2009) Mathan Twelve diabetes clinics (Italy).	Quantitative study.	To assess the utility of a telecare approach to diabetic pregnancy management.	T = 105 (GDM = 88; PGDM = 17) Mathan C = 130 (GDM = 115; PGDM = 15)	Interfacing device allows conversion of blood glucose values recorded by the glucometer into audio tones and transmission of glycaemic data through a telephone receiver. Individuals can add a voice message to provide physicians with any further details. Physicians can download and analyse data and record prescriptions to be sent to women through the interfacing device. An alert via text message is provided both to physicians and women when their messages were received by the others.	Standard care.	Glycaemic management. Mathan Maternal and fetal outcomes.	Illness acceptance. Mathan Diabetes-related distress. Mathan Health-related quality of life. Mathan Depressive symptoms. Mathan Person satisfaction with the telemedicine system.	Center for Epidemiologic Studies - Depression scale. Mathan 36-Item Short Form Health Survey (SF-36). Mathan Diabetes Health Stress. Mathan Diabetes Health Distress.	NA	NA
Given et al. (2015) Mathan Two antenatal diabetes clinics (Ireland).	Quantitative study; RCT.	To evaluate the effectiveness of a commercial telemedicine system as an integrative approach to standard care of GDM women and to assess its acceptability and feasibility among women and healthcare professionals.	T = 24 Mathan C = 26	The telemedicine approach was provided as an addition to usual care. The telemedicine system used was commercially available and included a set of scales, a blood pressure monitor, a blood glucose meter, and a telemedicine hub. The latter allows the collection of data, its transmission to women's healthcare professionals and reminds women to attend the telemedicine session (once per week). Moreover, a website was available where women could review their own data. If any concerns emerged, healthcare professionals contacted women by telephone to discuss any changes (e.g., medication, diet) or to arrange a face-to-face appointment.	Standard care.	Antenatal and neonatal outcomes. Mathan Time to review a person in the clinic or using the telemedicine system.	Women's satisfaction with the telemedicine system.	Adapted version of the Telemedicine satisfaction and usefulness questionnaire + semi-structured interview.	Healthcare professionals' perception of benefits for their workload and for persons' care.	Semi-structured interview.
Harrison et al. (2017) Mathan Kaiser Permanente Southern California (California).	Qualitative study.	To assess the acceptability of a hypothetical telemedicine-augmented GDM care protocol.	T = 10	The intervention combines remote visits and telemonitoring with standard face-to-face visits.	NA	NA	Perception of remote visits alternated with face-to-face visits. Mathan Confidence and comfort with the programme. Mathan Safety concerns related to the programme.	Qualitative interview.	NA	NA
Hirst et al. (2015) Mathan Oxford University Hospital National Health Service (England).	Qualitative study.	To assess individuals' satisfaction of care using GDm-health, a smartphone application-based blood glucose monitoring system.	T = 52	The application allows to collect monitoring data (blood glucose reading, medication and meals) and transmits it via the 3G network to a website hosted within the National Health Service. Communication between women and the diabetes midwife occurs, if required, by means of SMS or phone call.	NA	NA	Maternal satisfaction with care integrated using the app.	Oxford Maternity Diabetes Treatment Satisfaction Questionnaire (OMDTSQ).	NA	NA
Homko et al. (2007) Mathan Temple University Hospital (Pennsylvania).	Quantitative study; RCT.	To assess whether an Internet-based system could improve diabetes self-efficacy, empowering women with GDM to take an active role in their process of care.	T = 32 Mathan C = 25	The Internet-based system (ITSMyHealthfile) comprises an Internet server and a database. It allows persons to send health data (blood glucose and other relevant data) to their healthcare provider and to receive back therapeutic recommendations.	Standard care.	Glycaemic management.Mathan Neonatal outcomes.Mathan Use of the system for monitoring data transmission.	Self-efficacy in management of GDM.	Diabetes Empowerment Scale (DES).	NA	NA
Jo and Park (2016) Mathan NR (Korea).	Quantitative study.	To develop and test user acceptance of a smartphone application able to provide tailored support for GDM self-management.	T = 60 (acceptability was rated by 22 participants)	The application provides tailored recommendations based on an initial assessment of a person's lifestyle and clinical data. Recommendations are displayed in a number of screens and included management in the following domains: diet, blood glucose, physical activity, ketone, body weight.	NA	NA	Behavior intention to use the smartphone application. Mathan Intrinsic motivation. Mathan Perceived ease of use. Mathan Perceived usefulness of the app.	Wilson and Lankton's model of patients' acceptance of provider-delivered e-health.Mathan Technology Acceptance Model.	NA	NA
Kruger et al. (2003) Mathan Endocrinology and Metabolism Clinic, Henry Ford Health System (Michigan).	Quantitative study.	To examine the effect of modem transmission of blood glucose data on consultation time, clinic work-flow efficiency and accuracy of data received for women with GDM. Moreover, it aimed to assess women's and health care professionals' satisfaction.	T = 18 Mathan C = 20	Women with GDM followed the established standard care of the clinic, but they are requested to transmit blood glucose data using the Acculink Modem.	Standard care. It requires that women perform blood glucose measurements five times per day and report values to the clinic by phone weekly.	Accuracy of blood glucose data. Mathan Telephone consultation time. Mathan Length of clinic visit.	Women satisfaction.	Satisfaction questionnaire.	Satisfaction with the blood glucose meter and with the modem.	Satisfaction questionnaire.
Rigla et al. (2018) Mathan Hospital (Spain).	Quantitative study.	To evaluate the feasibility and acceptability of a mobile decision support system (MobiGuide) for GDM.	T = 20 Mathan C* = 247	The monitoring system included a smartphone, a glucometer device, and a blood pressure monitor. The smartphone was provided with a software designed for data collection, messages, detection of physical activity and ketonuria determination. Women were asked to download blood glucose values every 3 days, to measure blood pressure twice a week, to use the software for physical activity monitoring, and to inform on ketonuria status and diet behaviors.	Standard care.	Women's behavior related to self-monitoring of blood glucose (four times a day). Mathan Metabolic and perinatal outcomes.	Women's satisfaction with the system.	*Ad hoc* questionnaire for measuring satisfaction.	NA	NA
Wickramasinghe and Gururajan (2016) Mathan A private hospital (Australia).	Quali-quantitative study.	To assess the usability and acceptability of a pervasive mobile technology system for monitoring of women with GDM.	T = 10	The technology (Inet) is a software solution developed for facilitating woman–clinician interaction in the management of blood glucose monitoring. It comprises the use of a mobile phone and a web-based system. This technology solution was delivered alone or combined with standard care.	NA	Compliance with self-monitoring of blood glucose.	Women's satisfaction with standard care, the technology system and standard care combined with the technology system.	*Ad hoc* questionnaires.	Clinical care team (obstetrician, endocrinologist and diabetic educator) opinion of the technology compared to standard techniques.	*Ad hoc* questionnaire.

*C, Control group; GDM, Gestational Diabetes Mellitus; NA, Not Available; NR, Not Reported; PGDM, Pregestational Diabetes Mellitus; T, Telemedicine group*.

* *Cohort group who had been followed up for the 3 years prior to this study*.

Four studies evaluated patients' compliance in correlation with telemedicine care. In all, women using telemedicine systems were more compliant than women in traditional care groups.

The first study aimed at evaluating compliance was the work of Bartholomew et al. (2015). They tested a cell phone Internet technology for the management of hyperglycaemia during pregnancy. As an outcome, the authors found a statistically significant difference: women in the intervention group showed collaboration with clinicians, sharing their recommendations more than women who used a voicemail system (*p* = 0.048).

In line with the above results, Wickramasinghe and Gururajan (2016) designed a pilot study to assess the feasibility of a pervasive technology solution for GDM women. Feasibility was meant as compliance, women and clinicians' satisfaction and glycaemic management. According to questionnaires and interview results, compliance, and participation were higher in the intervention group.

Another medical and technical evaluation of a telemedicine system for women with GDM was presented by Bromuri et al. (2016). Its results indicated that the telemedicine group recorded more blood values than did the standard protocol group (*p* < 0.001).

The last and most recent study is that of Caballero-Ruiz et al. (2017). They evaluated the compliance to daily blood glucose measures and the monitoring and frequency use of the system. The authors expected at least four measurements per day and a frequency of uploading of data every 3 days. The results indicated the women were compliant, showing to be in line with the clinicians' indications.

Four studies assessed the clinicians' acceptability of the technology used by means of questionnaires, focus groups, and semi-structured interviews (Kruger et al., 2003; Given et al., 2015; Bromuri et al., 2016; Wickramasinghe and Gururajan, 2016). The telemedicine technology solution received positive feedback from the clinical staff in all studies. Effects were perceived both for their workload and for persons' care. The only disadvantage of telemedicine reported by healthcare providers was that the opportunity to talk to patients directly was lost (Given et al., 2015; Wickramasinghe and Gururajan, 2016).

Studies were categorized based on the psychological dimension assessed. A first paragraph summarizes existing research findings on empowerment or self-efficacy, and a second on satisfaction.

#### Telemedicine in GDM: Empowerment and Self-Efficacy

Homko et al. (2007) elaborated on a telemedicine system that gave users the possibility to use a link to the American Diabetes Association website where they could find educational material; indeed, disseminating information through technology can foster the system's credibility and in turn affect persons' commitment and self-efficacy (Fogg, 2003; Fantinelli and Cortini, 2018). The study by Homko et al. (2007) is the only one to evaluate self-efficacy and empowerment, adopting the Diabetes Empowerment Scale (DES) (Anderson et al., 2000). They compared the levels of empowerment and glucose of women in the intervention group with subjects following traditional care. The intervention consisted of a web-based solution capable of facilitating communication between persons with diabetes and clinicians. The authors postulated that women should feel more empowered and this feeling would affect their self-efficacy, fostering better glycaemia management, and pregnancy outcomes. The results showed that women in the intervention group revealed higher feelings of diabetes self-efficacy than did subjects in the control group (*p* = 0.053). However, at the same time, women in the intervention group needed more insulin therapy compared to the control group (*p* < 0.05). Therefore, the initial hypothesis was not completely confirmed. Homko et al. (2007) stated that this could be related to the short period of time of the intervention.

The research by Carolan-Olah et al. (2015) can be included in the empowerment section even if the authors did not directly evaluate empowerment. Nevertheless, the theoretical framework of the study was adult learning theory; the aim was to increase the women's empowerment thanks to the information and the knowledge concerning GDM that were offered by the telemedicine system. The results showed that the system can be more valuable for women with a lower educational level and it is a good way to foster changes in health-related attitudes and behaviors.

#### Telemedicine in GDM and Satisfaction

We screened twelve papers for what concerns the evaluation of satisfaction, which has been investigated in many different ways and in each sample there were positive outcomes.

Satisfaction was first measured in 2003 by Kruger et al. (2003). They evaluated the effects of modem transmission of blood glucose data on telephone consultation time, clinic work-flow, persons with GDM and health care providers' satisfaction. There were no significant differences between the two groups (*p* = 0.71), but both clinicians and women were satisfied with the telemedicine system. It is worth noting that health care providers perceived an improvement in work-flow efficiency, which could lead to a higher job satisfaction. Clinicians' job satisfaction can affect the quality of care and their relationships with persons with GDM and, in turn, positively affect the women's engagement (Graffigna et al., 2017).

Dalfrà et al. (2009) evaluated several psychological dimensions in relation to telemedicine systems: diabetes-related distress, quality of life, depressive symptoms and persons' satisfaction with the system. Women declared being satisfied with the system, indeed they appreciated the possibility to contact the physician whenever they needed.

The study by Hirst et al. (2015) was original from a methodological point of view because they created the Oxford Maternity Diabetes Treatment Satisfaction Questionnaire (OMDTSQ), since there were no questionnaires including satisfaction with technology in gestational diabetes. The results indicated that the women were very satisfied with the telemedicine system and they could perceive the support provided by the application.

In a subsequent study (Given et al., 2015), a more general questionnaire was implemented: The Telemedicine Satisfaction and Usefulness Questionnaire (Bakken et al., 2006). Women declared satisfaction with the telemedicine system, mentioning the time saving as the most common perceived advantage, as also stated by women in a subsequent study (Harrison et al., 2017). However, on the other side, both women and clinicians were worried about the loss of face-to-face relationships. Indeed, there is a risk related to the loss of empathy in the telemedicine communication which is an already known consequence (Liu et al., 2007).

Bartholomew et al. (2015), in addition to individuals' compliance, evaluated women's satisfaction with blood sugar monitoring testing an Internet-based technology. They also implemented a reminder feature consisting of text messaging to encourage individuals or as a reward for good practices. Most participants preferred the telemedicine system and were more satisfied (*p* < 0.001), particularly with the ease of use, time management, motivation, personalisation and self-efficacy. In line with previous results, Wickramasinghe and Gururajan (2016) also detected a good satisfaction rate related to the use of a pervasive technology solution for GDM; it was a pilot study conducted with a qualitative design, and the authors planned to test it further in a clinical trial.

In the study by Caballero-Ruiz et al. (2017), the authors created an *ad hoc* questionnaire aimed at evaluating a system's usability, usefulness, and trustworthiness. The results indicated that the women were highly satisfied, considered it useful and trusted in the care provided.

García-Sáez et al. (2014) described a telemedicine device in 2014, called MobiGuide, to support clinicians' knowledge management and persons' decision making; the innovation resides in the personalized feedback for women with GDM, with customization based on meals or blood glucose data. The MobiGuide system was subsequently evaluated and described in another study (Rigla et al., 2018). Results showed a high level of satisfaction with the system through an *ad hoc* questionnaire.

Another study that described a telemedicine approach with persuasive features is the Jo and Park (2016) research on a smartphone application. This study was the first attempt to provide tailored recommendations for GDM management based on data entered by patients. They measured acceptance of the app through four constructs: behavioral intention to use; intrinsic motivation; perceived ease of use; perceived usefulness. The survey showed positive results in terms of behavioral intention to use and perceived usefulness reported by participants.

## Discussion

To the best of our knowledge, this is the first systematic review that analyses the effects of telemedicine in GDM with respect to psychological dimensions. Our review aimed at contributing to the expansion of knowledge concerning women with GDM and the use of telemedicine. We were interested in investigating the influence of telemedicine systems on engagement, self-efficacy, empowerment, and satisfaction with care.

Overall, all the selected studies displayed potential sources of methodological bias. Our systematic review of quantitative and qualitative studies on telemedicine for GDM, especially when psychological dimensions were assessed, showed that good quality trials in this area were few in number and need further enhancement. This has been pointed out elsewhere (Rasekaba et al., 2015; Ming et al., 2016).

The results show that there are no studies evaluating engagement related to telemedicine in GDM and the most common observed significant results are associated with clinical outcomes, such as compliance, blood glucose levels and pregnancy outcomes. There were only 13 studies aimed at evaluating psychological variables related to the use of telemedicine systems in GDM: twelve evaluated women's perceptions and satisfaction with the telemedicine system with quantitative and qualitative methods, reporting consistent and positive results. One study considered quality of life and depression together with satisfaction through quantitative questionnaires. Concerning empowerment and self-efficacy, there are only preliminary findings reporting any improvements derived from using telemedicine approaches. Indeed, only one study evaluated self-efficacy.

According to the literature reviewed, it is possible to state that telemedicine use in GDM management has an expected positive impact. The most common positive outcome is related to the reduction in visit numbers, which in turn can aid more efficient clinician work and a better quality of life for pregnant women; in addition to other positive effects of telemedicine on women with GDM, as already observed by a recent systematic review (Marchetti et al., 2017). As already noted, telemedicine can also positively affect time and cost saving and this represents a valuable advantage since GDM is limited in time and women have a short time to know and accept their condition.

However, it is worth noting the negative feedback reported by clinicians. Some authors (Given et al., 2015) reported that the loss of face-to-face contact between patient and clinician was detrimental; healthcare practitioners had more confidence in defining and managing therapy when talking face-to-face with patients; the medium of technology was defined as a limitation. Furthermore, the use of telemedicine implies a good technological literacy for both patients and physicians; indeed, in several studies this element represented an eligibility criterion.

We can state that, compared to previous systematic reviews, the relevance of our results is related to specific attention only on GDM and to the openness to relevant variables such as empowerment, self-efficacy, and satisfaction other than clinical outcomes. It is quite surprising that there is just one study (Carolan-Olah et al., 2015) describing a telemedicine system that also includes some combined information related to fetal health or growth. More specifically, the study described a website designed to provide information in order to improve GDM knowledge. However, given that GDM is such a delicate and transitory situation for the woman, she could probably be more motivated to adhere to the therapy or to feel engaged just thinking of the baby's future well-being. Therefore, we hypothesize that this specific combination of interests can be further expanded by different perspectives, such as psychological, clinical, and technological. With regard to our outcomes, we also assume that a positive dimension of telemedicine that is worth implementing is the information providing feature, as worthwhile information can enhance women's engagement and acceptance of their condition.

In addition to these considerations, it must be said that as far as we are concerned, there are no telemedicine systems providing a supportive and monitoring role after delivery. Indeed, all the experts and scientific societies clearly recommend a post-partum follow up to prevent future chronic conditions for the mother or the baby (Hod et al., 2015, 2017; American Diabetes Association, 2018). Following these significant indications, it could be possible to design a telemedicine system capable of producing reminders for medical visits after childbirth.

Maybe, mobile health applications are the most frequent current telemedicine implementations. The Food and Drug Administration recently elaborated on recommendations concerning application development, in order to make them respectful of persons and therapy (US Food Drug Administration, 2018). In line with the concept of knowledge and informative empowerment, some relevant applications exist. Accordingly, the Italian Pregnancy Study Group (Italian Diabetes in Pregnancy Study Group AMD-SID, 2017) recently designed the application MySweetGestation, which is a purely practical guide about the risk of diabetes for women who are planning a pregnancy or are pregnant. This kind of application aims at providing reliable and scientifically validated knowledge to users and at offering reassurance about chronic condition management, thus, tackling the information overload that can impede information processing (Kim et al., 2007). Moreover, MySweetGestation offers two sections: one dedicated to women, the second dedicated to clinicians who wish to expand their knowledge about GDM.

An aspect deserving further research is clinicians' point of view. For example, some authors have investigated expert diabetologists' acceptability of a telemedicine system, finding that a knowledge-based system could produce advice for diabetes management starting from the person's self-monitored data (Hernando et al., 2000). Telemedicine would reduce the workload of healthcare providers allowing them to spend more time with each patient and would be beneficial for patients. However, qualitative data highlighted that their approval tends to be more measured than that of patients (Whitten and Love, 2005; Given et al., 2015). Health professionals' perceived impact and attitude toward telemedicine care may influence patients' acceptance, helping overcome barriers. A deeper investigation of the acceptability by health care professionals should be considered for future studies. This area of research could provide a contribution to a broader understanding of the effects of technology in the management of GDM.

The use of a telemedicine system surely affects health care providers jobs as well; the consequences for job satisfaction, commitment and job efficiency can in turn affect persons' engagement, as already hypothesized by Graffigna et al. (2017). However, important questions regarding this topic still remain open, especially those related, on the one hand, to the acceptability of such devices and on the other, to the analysis of potential differences related to aging. Specifically, it seems to us mandatory to further investigate some other challenges, for example how work ability in medicine is changing with the implementation of telemedicine, as it has already been studied with other occupations, like for example teaching (Converso et al., 2018), where work ability has changed dramatically in recent years.

Furthermore, it must be highlighted that no specific studies on telemedicine, GDM and engagement were found. An important reflection is that women during pregnancy, with GDM particularly, need a complete periodic clinical control that unfortunately telemedicine does not offer. In recent years, some studies have investigated this association in persons living with diabetes, reporting interesting results: an exploratory study revealed that individuals with type 1 and 2 diabetes manifested a higher collaboration with clinicians using the m-health system for 4 weeks (Fioravanti et al., 2015). Another interesting result is described by Tang et al. (2013). In their study persons with type 2 diabetes using an eHealth platform reported higher levels of engagement compared to persons in the control group. Engagement was evaluated in terms of satisfaction and active participation in communication with the health provider. According to a recent literature review concerning the relation between e-health and persons' engagement, the engagement dimensions evaluated were those related to behavior and emotion (Barello et al., 2016). Therefore, exploring the effect of behavioral, emotional and cognitive engagement on GDM outcomes is a noteworthy angle of research to be further expanded and analyzed in depth. Furthermore, it is important to underline that GDM can be considered an opportunity to start a different lifestyle in order to have optimal blood glucose and to change potential unhealthy habits.

To conclude, as already noted by Chilelli et al. (2014), one aim of telemedicine implementation should be the enhancement of chronic condition management by women, thus making their lifestyles more compatible with diabetes management. However, it is still not fully demonstrated what kind of telemedicine can effectively support GDM management in terms of clinical outcomes considering both women's and clinicians' points of view. Related to this line of research, future studies should consider examining the mediating and/or moderating roles of psychological dimensions (e.g., self-efficacy, empowerment) in the impact of telemedicine on clinical outcomes of pregnancies complicated by GDM.

## Author Contributions

SF, DM, MCV, and EV contributed to the conception of this review. SF and DM performed the literature search and wrote the first draft of the manuscript. MCV and EV revised the first draft of the manuscript. All authors contributed to the subsequent drafting and rewriting of the manuscript. All authors approved the final version of the manuscript.

### Conflict of Interest Statement

The authors declare that the research was conducted in the absence of any commercial or financial relationships that could be construed as a potential conflict of interest.
